# Prevalence of late cutaneous reactivity to metabisulfites in patients with and without chronic eczema in Argentina

**DOI:** 10.1186/1939-4551-8-S1-A71

**Published:** 2015-04-08

**Authors:** Ledit Ardusso, Jorge Molinas, Victor Espirito Santo Cunha, Tielli Magnus, Ruppert Ludwig Hahnstadt

**Affiliations:** 1 Medical Sciences University. National University of Rosario, Argentina; 2 University of the Center Educational Latino Americano, Argentina; 3 FDA Allergenic, Brazil

## Background

Sulfites are used as preservatives and antioxidants in the cosmetic, pharmaceutical and food industry. The prevalence of sensitization caused by sulfites in Argentina is unknown.

## Methods

Twenty two participants, 14 males and 8 females (mean age 31 years old), without eczema (Group 1) and 20 patients, 3 males and 17 females (mean age 35 years old), with chronic eczema (Group 2), living in Rosario, Argentina, were patch tested (PT) with sodium metabisulfite (MBS – CAS: 7681-57-4) 1% in petrolatum and with Brazilian Contact Standard Series (FDA Allergenic, Brazil). For the test, Finn Chambers (SmartPractice, USA) with 8mm (standard series) and 12mm (MBS) of diameter were used and the patch testing was performed according to the International Contact Dermatitis Research Group criteria (2 days of occlusion, readings on D2 and D4, using a score from + to +++). Chi-squared test was used to examine whether there was a significantly statistical difference between positive reactions frequencies observed in two groups. The significance level for group differences was set at *p*< 0.05. The software used for this analysis was the GraphPad Prism version 6.

## Results

Three participants (13.6%) of Group 1 and four (20%) of Group 2 presented positive reactions to MBS. There was no statistical difference between groups (*p*=0.58). All participants of group 2 presented at least one positive reaction to substances of the Brazilian Contact Dermatitis Standard Series (FDA Allergenic) while no participants from Grupo 1 reacted to it. None of the patients with MBS PT (+) and/or (-) had a suspected history of symptoms elicitation after the consumption of food and/or beverages with MBS as additive.

## Conclusions

We concluded that MBS 1% in petrolatum is suitable for patch test but the relevance of positive reaction to MBS 1% has to be better explained before its use in standard series.

